# Molecular profiling and clinical implications of patients with acute myeloid leukemia and extramedullary manifestations

**DOI:** 10.1186/s13045-022-01267-7

**Published:** 2022-05-13

**Authors:** Jan-Niklas Eckardt, Friedrich Stölzel, Desiree Kunadt, Christoph Röllig, Sebastian Stasik, Lisa Wagenführ, Korinna Jöhrens, Friederike Kuithan, Alwin Krämer, Sebastian Scholl, Andreas Hochhaus, Martina Crysandt, Tim H. Brümmendorf, Ralph Naumann, Björn Steffen, Volker Kunzmann, Hermann Einsele, Markus Schaich, Andreas Burchert, Andreas Neubauer, Kerstin Schäfer-Eckart, Christoph Schliemann, Stefan W. Krause, Regina Herbst, Mathias Hänel, Maher Hanoun, Ulrich Kaiser, Martin Kaufmann, Zdenek Rácil, Jiri Mayer, Frank Kroschinsky, Wolfgang E. Berdel, Gerhard Ehninger, Hubert Serve, Carsten Müller-Tidow, Uwe Platzbecker, Claudia D. Baldus, Johannes Schetelig, Martin Bornhäuser, Christian Thiede, Jan Moritz Middeke

**Affiliations:** 1 Department of Internal Medicine I, University Hospital Carl Gustav Carus, Fetscherstraße 74, 01307 Dresden, Saxony, Germany; 2 Department of Pathology, University Hospital Carl Gustav Carus, Dresden, Germany; 3 Medical Care Center, University Hospital Carl Gustav Carus, Dresden, Germany; 4 German Cancer Research Center (DKFZ) and Medical Clinic V, University Hospital Heidelberg, Heidelberg, Germany; 5 Department of Internal Medicine II, Jena University Hospital, Jena, Germany; 6 Department of Hematology, Oncology, Hemostaseology, and Cell Therapy, University Hospital RWTH Aachen, Aachen, Germany; 7 Medical Clinic III, St. Marien-Hospital Siegen, Siegen, Germany; 8 Medical Clinic II, University Hospital Frankfurt, Frankfurt (Main), Germany; 9 Medical Clinic and Policlinic II, University Hospital Würzburg, Würzburg, Germany; 10 Department of Hematology, Oncology and Palliative Care, Rems-Murr-Hospital Winnenden, Winnenden, Germany; 11 Department of Hematology, Oncology and Immunology, Philipps-University-Marburg, Marburg, Germany; 12 Department of Internal Medicine V, Paracelsus Medizinische Privatuniversität and University Hospital Nuremberg, Nuremberg, Germany; 13 Department of Medicine A, University Hospital Münster, Münster, Germany; 14 Medical Clinic V, University Hospital Erlangen, Erlangen, Germany; 15 Medical Clinic III, Chemnitz Hospital AG, Chemnitz, Germany; 16 Department of Hematology, University Hospital Essen, Essen, Germany; 17 Medical Clinic II, St. Bernward Hospital, Hildesheim, Germany; 18 Department of Hematology, Oncology and Palliative Care, Robert-Bosch-Hospital, Stuttgart, Germany; 19 Department of Internal Medicine, Hematology and Oncology, Masaryk University Hospital, Brno, Czech Republic; 20 Medical Clinic I Hematology and Celltherapy, University Hospital Leipzig, Leipzig, Germany; 21 Department of Internal Medicine, University Hospital Kiel, Kiel, Germany; 22 DKMS Clinical Trials Unit, Dresden, Germany; 23 German Consortium for Translational Cancer Research DKFZ, Heidelberg, Germany; 24 National Center for Tumor Diseases (NCT), Dresden, Germany

**Keywords:** Acute myeloid leukemia, Extramedullary, Leukemia cutis, Chloroma, Myeloid sarcoma

## Abstract

**Background:**

Extramedullary manifestations (EM) are rare in acute myeloid leukemia (AML) and their impact on clinical outcomes is controversially discussed.

**Methods:**

We retrospectively analyzed a large multi-center cohort of 1583 newly diagnosed AML patients, of whom 225 (14.21%) had EM.

**Results:**

AML patients with EM presented with significantly higher counts of white blood cells (*p* < 0.0001), peripheral blood blasts (*p* < 0.0001), bone marrow blasts (*p* = 0.019), and LDH (*p* < 0.0001). Regarding molecular genetics, EM AML was associated with mutations of *NPM1* (OR: 1.66, *p* < 0.001), *FLT3*-ITD (OR: 1.72, *p* < 0.001) and *PTPN11* (OR: 2.46, *p* < 0.001). With regard to clinical outcomes, EM AML patients were less likely to achieve complete remissions (OR: 0.62, *p* = 0.004), and had a higher early death rate (OR: 2.23, *p* = 0.003). Multivariable analysis revealed EM as an independent risk factor for reduced overall survival (hazard ratio [HR]: 1.43, *p* < 0.001), however, for patients who received allogeneic hematopoietic cell transplantation (HCT) survival did not differ. For patients bearing EM AML, multivariable analysis unveiled mutated *TP53* and *IKZF1* as independent risk factors for reduced event-free (HR: 4.45, *p* < 0.001, and HR: 2.05, *p* = 0.044, respectively) and overall survival (HR: 2.48, *p* = 0.026, and HR: 2.63, *p* = 0.008, respectively).

**Conclusion:**

Our analysis represents one of the largest cohorts of EM AML and establishes key molecular markers linked to EM, providing new evidence that EM is associated with adverse risk in AML and may warrant allogeneic HCT in eligible patients with EM.

**Supplementary Information:**

The online version contains supplementary material available at 10.1186/s13045-022-01267-7.

## Background

Extramedullary manifestation (EM) of infiltrating clonal blast populations in a variety of organs and tissues in acute myeloid leukemia (AML) is defined as a distinct entity in the 2016 WHO classification of myeloid neoplasms, myeloid sarcoma [1], (or synonymously granulocytic sarcoma [2] or chloroma [3]) and can either present concurrently with bone marrow and/or peripheral blood involvement, or isolated, and in some cases even antecedent to bone marrow involvement or relapse [4]. While manifestations can be found in a wide variety of organs, most frequent locations include lesions in the connective tissues, intestinal organs and the skin, where EM AML is often referred to as leukemia cutis [4, 5].

Previous reports have estimated EM to be present in 2–9% of AML cases [6–8]. However, the recent PET-AML trial has reported a frequency of 17% in newly diagnosed AML patients [9]. Nevertheless, genetic events and molecular mechanisms that lead to the formation of EM in AML and the impact on clinical outcomes are not well understood and previous studies are commonly confined to small samples or case series, often with controversial results [4, 10, 11].

We here present a large multi-center cohort of newly diagnosed and intensively treated AML patients to compare cytogenetic and molecular profiles as well as clinical presentations and outcomes between AML presenting with concurrent EM and non-EM AML.

## Methods

### Patient cohort

We analyzed 1583 adult patients with newly diagnosed AML from previous multi-center clinical trials (AML96 [12], AML2003 [13], AML60+ [14], and SORAML [15]) and the multi-center German Study Alliance Leukemia (SAL) AML registry (NCT03188874). Eligibility was determined by age ≥ 18 years, diagnosis of AML according to WHO criteria [1], curative treatment approach with intensive induction therapy and available biomaterial at initial diagnosis. AML without antecedent history of malignancy or radio-/chemotherapy was defined as de novo, while AML not fulfilling these criteria or with prior history of myeloid neoplasms was defined as secondary AML (sAML) and AML with prior radio- and/or chemotherapy for the treatment of non-myeloid malignancies was defined as therapy-associated AML (tAML). Early death (ED_30_) was defined as death from any cause within 30 days after initial diagnosis. EM AML status was determined at baseline by clinical examination for the entire cohort. Additional histopathologic confirmation of EM in the respective organ was available in 38 cases. The investigation was carried out under the auspices of the SAL registry and received approval of the Institutional Review Board of the Technical University Dresden (EK 98032010). Written informed consent was obtained from all participants according to the Declaration of Helsinki.

### Molecular and cytogenetic profiling

All studies were performed on pre-treatment bone marrow aspirates or peripheral blood. Cytogenetic profiling was done using standard techniques for chromosome banding and fluorescence in situ hybridization (FISH). Molecular profiling was done using high resolution fragment analysis for *FLT3*-ITD [16], *NPM1* [17] or *CEBPA* [18]. For additional alterations, characterization was done using the TruSight Myeloid assay (Illumina, San Diego, CA, USA) as described in detail previously [19, 20]. Briefly, the panel targets 54 genes that are frequently mutated in myeloid neoplasms (Additional file 1: Table S1). Genomic DNA was isolated using the DNeasy Blood and Tissue Kit (Qiagen, Hilden, Germany) and quantified using the NanoDrop (ThermoFisher, Waltham, MA, USA) spectrophotometer. For each reaction, 50 ng of genomic DNA was used. Samples were sequenced paired-end on a NextSeq (150 bp PE) or MiSeq (300 bp PE) NGS-instrument (Illumina, San Diego, CA, USA). A 5% variant allele frequency (VAF) mutation calling cut-off was used.

### Statistical analysis

Statistical analysis was performed and visualizations were created using STATA BE 16.0 (Stata Corp, College Station, TX, USA) and R 4.1.2 (R Foundation, Vienna, Austria). Categorical variables were compared using the two-sided Fisher’s exact test. Normality was evaluated using the Shapiro–Wilk test. If the assumption of normality was met, continuous variables between two groups were analyzed using the two-sided unpaired t-test. If the assumption of normality was violated, continuous variables between two groups were analyzed using the Wilcoxon rank sum test. Univariable analysis to test for the effect of different mutations on EM AML status as well as EM AML’s impact on ED_30_ and CR was carried out using logistic regression. For survival analysis including the evaluation of event-free survival (EFS), relapse-free survival (RFS) and overall survival (OS), the Kaplan–Meier method and the log-rank test were used. For univariable and multivariable analysis of prognostic markers, Cox-proportional hazard models were used. For both odds ratios (OR) and hazard ratios (HR), 95%-confidence intervals (95%-CI) are reported. Statistical significance was determined using a significance level α of 0.05.

## Results

**Fig. 1:**
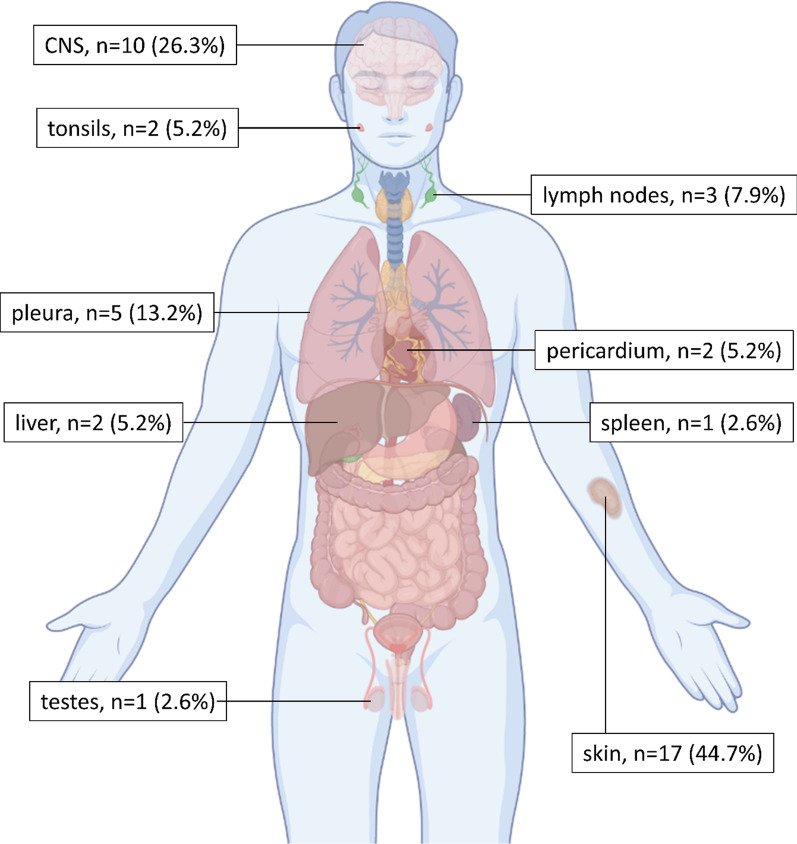
Distribution of histopathologically confirmed extramedullary manifestations. For 38 out of 225 patients, biomaterial of the affected site was available for histopathological confirmation of extramedullary manifestations (EM). Most frequently, EM was found in the skin, central nervous system (CNS) and pleura. Three patients had two affected EM sites and one patient had three EM sites

EM AML was found in 225 of 1583 patients (14.2%), only two of whom presented with isolated extramedullary tissue infiltration, i.e., myeloid sarcoma [1], while the majority had concurrent bone marrow involvement. In 38 cases, additional biomaterial of the affected organ was available for histopathologic evaluation. Most frequently affected sites were skin (44.7%), central nervous system (26.3%), and pleura (13.2%). Figure 1 displays the distribution of histopathologically confirmed EM.

Regarding baseline patient characteristics (Table 1) we did not find differences in occurrence of EM AML between male or female patients (*p* = 0.773), de novo, sAML or tAML (*p* = 0.6587), or regarding age (*p* = 0.765). Patients with EM AML compared to patients without EM AML had significantly higher white blood cell (WBC) count (*p* < 0.0001), higher LDH (*p* < 0.0001), peripheral blood blast (PBB) counts (*p* < 0.0001) and bone marrow blast (BMB) counts (*p* = 0.019) while hemoglobin levels and platelet counts did not differ. Every increase of WBC by 5*10^9^/l led to an increase in the odds of EM AML of 0.005 (Additional file 1: Fig. S1A) and every one-percent increase in BMB (Additional file 1: Fig. S1B) and PBB (Additional file 1: Fig. S1C) counts led to an increase in the odds of EM AML of 0.01 for both. For LDH, every increase by 50 U/l led to an increase in the odds of EM AML by 0.002 (Additional file 1: Fig. S1D).

**Table 1:** Baseline patient characteristics

Parameter	Non-EM AML	EM AML	*p*
n/N (%)	1358/1583 (85.79)	225/1583 (14.21)	
Age (years), median (IQR)	53 (42–60)	53 (42–61)	0.7652
Sex, n (%)			0.7730
Female	656 (48.31)	106 (47.11)	
Male	702 (51.69)	119 (52.89)	
Disease status, n (%)			0.6587
De novo	1154 (84.98)	196 (87.11)	
sAML	143 (10.53)	21 (9.33)	
tAML	45 (3.31)	7 (3.11)	
ELN-Risk 2017, n (%)			0.0817
Favorable	423 (31.15)	81 (36.0)	
Intermediate	412 (30.34)	77 (34.22)	
Adverse	425 (31.30)	56 (24.89)	
Complex karyotype, n (%)			0.535
No	1106 (81.44)	201 (89.33)	
Yes	122 (8.93)	18 (8.0)	
Normal karyotype, n (%)			0.504
No	535 (39.40)	88 (39.11)	
Yes	727 (53.53)	129 (57.33)	
Laboratory, median (IQR)			
WBC (10^9^/l)	14.5 (3.3–47.2)	32.24 (11.1–87.0)	** < 0.0001**
HB (mmol/l)	5.9 (5.0–7.1)	5.9 (5.1–7.0)	0.5814
PLT (10^9^/l)	52 (28–96.5)	53 (28–94)	0.8999
LDH (U/l)	429 (260–724)	605 (462–1008)	** < 0.0001**
PBB (%)	33 (8–70)	55 (19–79.5)	** < 0.0001**
BMB (%)	63 (43–79)	68.5 (47.5–82.5)	**0.019**

Bold typing indicates statistical significance (*p* < 0.05)

*AML* acute myeloid leukemia, *sAML* secondary AML, *tAML* therapy-associated AML, *BMB* bone marrow blasts, *EM* extramedullary, *HB* hemoglobin, *IQR* interquartile range, *n*/*N* number, *PBB* peripheral blood blasts, *PLT* platelet count, *WBC* white blood cell count.

**Fig. 2:**
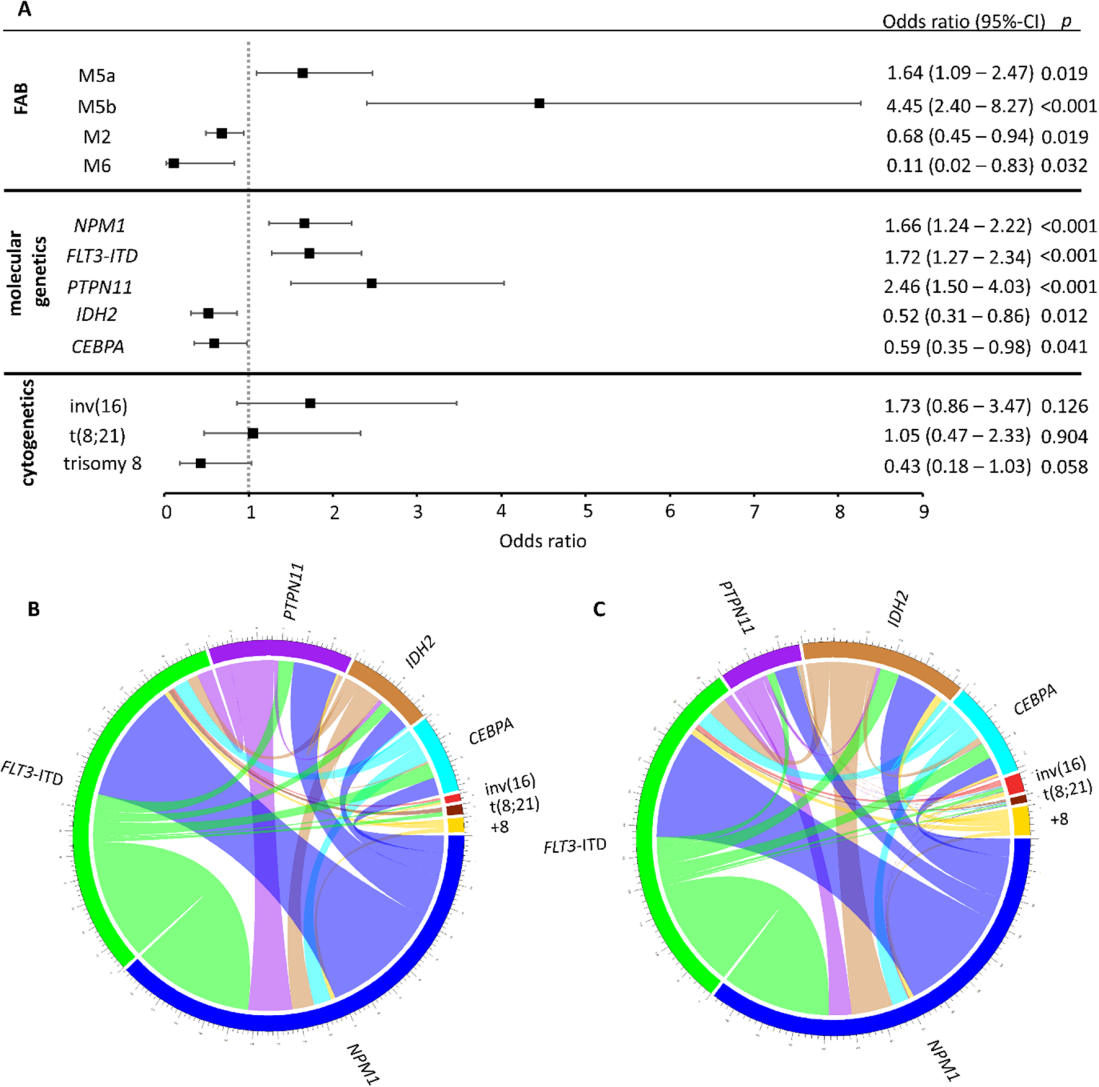
Parameters associated with extramedullary manifestations in AML. Logistic regression was used to obtain univariable odds ratios for presence or absence of extramedullary manifestations (EM) in AML patients (**A**). We found the presence of EM to be significantly associated with cytomorphologic phenotypes according to the French-American-British (FAB) classification. FAB-M5a and -M5b were associated with increased odds while -M2 and -M6 were associated with decreased odds. As for molecular genetics, mutations of *NPM1*, *FLT3*-ITD and *PTPN11* were associated with EM while *IDH2* and *CEBPA* were less likely to be associated with EM. Previous reports have suggested an association of inv [16], t(8;21) and trisomy 8 with EM, however in our analysis we did not find a statistically significant association. Molecular and cytogenetic interconnections of patients with (**B**) or without (**C**) EM AML are displayed

Regarding cytomorphologic subtypes according to the French-American-British (FAB) classification [21], we found significantly increased odds for the presence of EM AML for FAB-M5a (OR: 1.64 [95%-CI: 1.09–2.47], *p* = 0.019) and FAB-M5b (OR: 4.45 [95%-CI: 2.40–8.27], *p* < 0.001) while decreased odds were found for FAB-M2 (OR: 0.68 [95%-CI: 0.49–0.94], *p* = 0.019) and FAB-M6 (OR: 0.11 [95%-CI: 0.02–0.83], *p* = 0.032). As for molecular genetics, significantly increased odds of EM AML were found for *PTPN11* (OR: 2.46 [95%-CI: 1.50–4.03], *p* < 0.001), *NPM1* (OR: 1.66 [95%-CI:1.24–2.22], *p* < 0.001), and *FLT3*-ITD (OR: 1.72 [95%-CI: 1.27–2.34], *p* < 0.001) with an increase in *FLT3*-ITD ratio leading to a corresponding increase in the odds of EM AML (Additional file 1: Fig. S1E). However, mutations in *IDH2* (OR: 0.52 [95%-CI: 0.31–0.86], *p* = 0.012) and *CEBPA* (OR: 0.59 [95%-CI: 0.35–0.98], *p* = 0.041) were associated with a decrease in the odds of EM AML. No significant associations with EM AML were found for inv [16] (OR: 1.73 [95%-CI: 0.86–3.47], *p* = 0.126), t(8;21) (OR: 1.05 [95%-CI: 0.47–2.33], *p* = 0.904), trisomy 8 (OR: 0.43 [95%-CI: 0.18–1.03], *p* = 0.058), or other common cytogenetic aberrations. Odds ratios and confidence intervals for these parameters are summarized in Fig. 2.

**Fig. 3:**
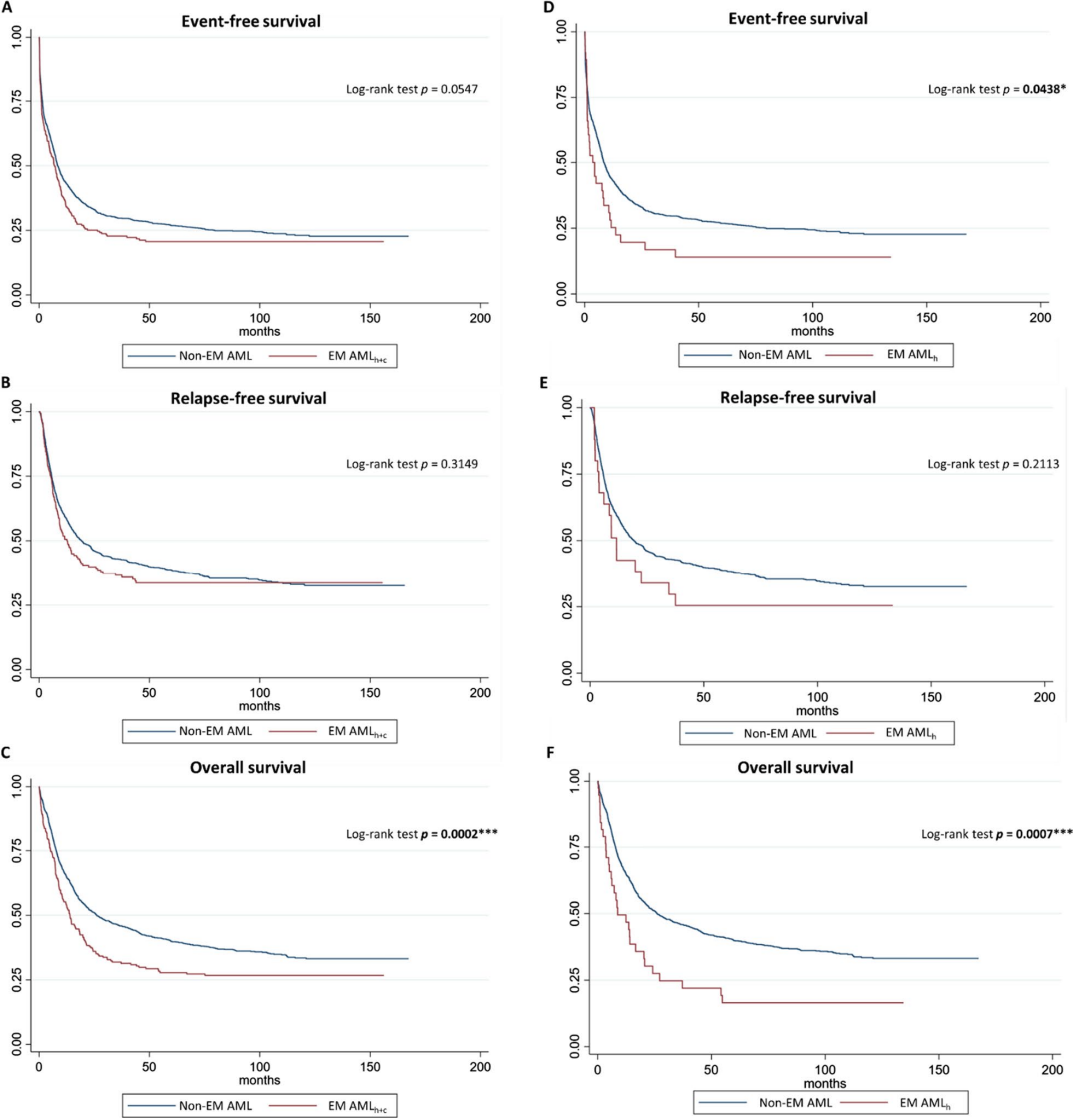
Impact of extramedullary manifestations on survival in acute myeloid leukemia. For the comparison between both clinically and histologically determined EM AML and non EM-AML, both event-free (**A**) and relapse-free survival (**B**) did not differ significantly while overall survival (**C**) was significantly reduced. When we focused only on histologically confirmed EM AML (n = 38) excluding cases for whom only clinical diagnosis of EM AML was available, we found that both event-free survival (**D**) as well as overall survival (**F**) were significantly reduced compared to AML patients without EM while relapse-free survival did not differ (**E**). Significance was determined at *α* = 0.05; * *p* < 0.05, ** *p* < 0.01, ***p < 0.001; EM AML_h+c_ = histologically and clinically diagnosed cases of EM AML (panel **A**–**C**); EML AML_h_ = only histologically confirmed cases of EM AML (panel **D**–**F**)

With respect to clinical outcomes, the odds of achieving CR with intensive induction therapy were significantly decreased for patients with EM AML (OR: 0.62 [95%-CI: 0.45–0.86], *p* = 0.004) while the odds of early death within 30 days after initial diagnosis were significantly increased (OR: 2.23 [95%-CI: 1.31–3.78], *p* = 0.003). However, relapse rates did not differ between patients with or without EM AML (OR: 1.01 [95%-CI: 0.76–1.35], *p* = 0.947). With regard to survival (Table 2), patients with EM AML compared to patients without EM did not differ significantly regarding median event-free survival (7.1 months [95%-CI: 4.8–9.1] vs. 8.4 months [95%-CI: 7.7–9.6], HR: 1.17 [95%-CI: 1.00–1.37], Cox regression *p* = 0.056, Fig. 3A) and median relapse-free survival (12.6 months [95%-CI: 9.3–18.4] vs. 19.3 months [95%-CI: 16.3–23.7], HR: 1.11 [95%-CI: 0.90–1.36], Cox regression *p* = 0.315, Fig. 3B). However, patients with EM AML showed significantly decreased median overall survival (14.0 months [95%-CI: 10.6–18.4] vs. 26.2 months [95%-CI: 22.4–32.6], HR: 1.38 [95%-CI: 1.16–1.63], Cox regression *p* < 0.001, Fig. 3C). In a multivariable model adjusting for ELN2017 risk groups and age (Additional file 1: Table S1), EM AML remained an independent marker of reduced OS (HR: 1.43 [95%-CI: 1.21–1.70], Cox regression *p* < 0.001).

**Table 2:** Survival times of patients with or without extramedullary manifestations in the entire patient cohort

Survival times	EM AML	Non-EM AML	Hazard ratio	Cox regression *p*-value
Event-free survival	7.1 [4.8–9.1]	8.4 [7.7–9.6]	1.17 [1.00–1.37]	0.056
Relapse-free survival	12.6 [9.3–13.4]	19.3 [16.3–23.7]	1.11 [0.90–1.36]	0.315
Overall survival	14.0 [10.6–18.4]	26.2 [22.4–32.6]	1.38 [1.16–1.63]	** < 0.001**

Survival times in months. Cox-proportional hazard models were used to obtain univariable hazard ratios. Brackets show 95%-confidence intervals. Statistically significant *p*-values are marked in bold

For 38 patients, additional biomaterial of the affected EM AML site was available. When we compared only these patients to non-EM AML patients and excluded those EM AML patients for whom only clinically diagnosed EM were available, the impact on outcome became more evident (Table 3). The odds ratio to achieve CR was significantly reduced for patients with histologically confirmed EM (OR: 0.47 [95%-CI: 0.24–0.92], *p* = 0.029) as well as both median EFS (3.6 months [95%-CI: 1.2–8.4], HR: 1.43 [95%-CI: 1.01–2.04], Cox regression *p* = 0.046, Fig. 3D) and OS (8.7 months [95%-CI: 5.1–20.3], HR: 1.84 [95%-CI: 1.29–2.64], Cox regression *p* < 0.001, Fig. 3F) while RFS did not differ (Fig. 3E). Again, in multivariable analysis adjusting for ELN2017 risk groups and age, EM remained an independent marker of reduced EFS (HR: 1.44 [95% CI: 1.01–2.05], Cox regression *p* = 0.042, Additional file 1: Table S2) as well as OS (HR: 1.67 [95% CI: 1.17–2.40], Cox regression *p* = 0.005, Additional file 1: Table S3).

**Table 3:** Survival times of patients with or without histologically confirmed extramedullary manifestations

Survival times	Hist. EM AML	Non-EM AML	Hazard ratio	Cox regression *p*-value
Event-free survival	3.6 [1.2–8.4]	8.4 [7.7–9.6]	1.43 [1.01–2.04]	**0.046**
Relapse-free survival	11.6 [3.9–34.6]	19.3 [16.3–23.7]	1.35 [0.84–2.15]	0.213
Overall survival	8.7 [5.1–20.3]	26.2 [22.4–32.6]	1.84 [1.29–2.64]	** < 0.001**

In comparison to Table 2, patients with only clinical diagnosis of extramedullary manifestations (EM) were excluded and only data for patients with histologically confirmed EM are shown (*n* = 38). Survival times in months. Cox-proportional hazard models were used to obtain univariable hazard ratios. Brackets show 95%-confidence intervals. Statistically significant* p*-values are marked in bold

**Fig. 4:**
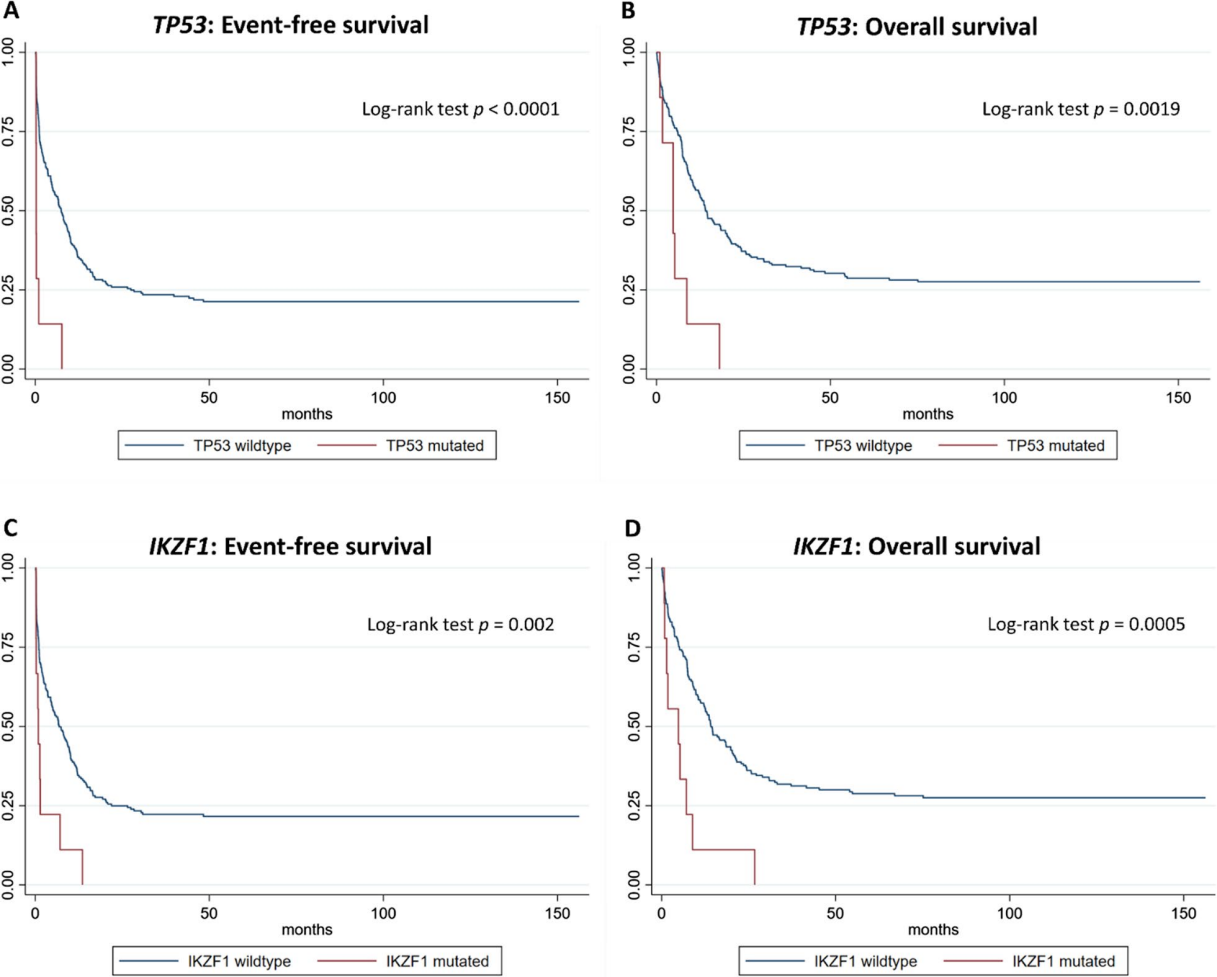
Risk factors in acute myeloid leukemia with extramedullary manifestations. Among AML patients bearing extramedullary manifestations (EM) mutations of *TP53* and *IKZF1* were rare (7/225 [3.1%] and 9/225 [4.0%], respectively). However, EM AML patients with TP53 mutations showed significantly decreased event-free (**A**) and overall survival (**B**). Likewise, EM AML patients with IKZF1 mutations also showed significantly decreased event-free (**C**) and overall survival (**D**)

For patients with EM AML (both clinical and histological), there were no significant differences regarding survival between either patients harboring normal or complex aberrant karyotypes. With respect to the impact of molecular genetics on survival in patients with EM AML, although mutations of *TP53* (7/225, 3.1%) and *IKZF1* (9/225, 4.0%) were rare, both alterations were significantly associated with decreased EFS and OS. In univariable analysis, patients with EM AML and mutated *TP53* compared to EM AML patients with wild-type *TP53* had decreased median EFS (0.26 months [95% CI: 0.23–0.97] vs. 7.43 months [95% CI: 5.00–9.40], HR: 4.77 [95% CI: 2.20–10.35], Cox regression *p* < 0.001, Fig. 4A) and OS (4.80 months [95% CI: 0.99–8.74] vs. 14.10 months [95% CI: 11.21–19.86], HR: 3.16 [95% CI: 1.47–6.82], Cox regression *p* = 0.003, Fig. 4B). In a multivariable analysis adjusting for age and ELN2017 risk groups, *TP53* remained an independent marker of reduced EFS (HR: 4.45 [95% CI: 1.94–10.20], Cox regression *p* < 0.001, Additional file 1: Table S4) and OS (HR: 2.48 [95% CI: 1.11–5.52], Cox regression *p* = 0.026 Additional file 1: Table S5). Reduced median EFS (0.85 months [95%-CI: 0.23–7.10] vs. 6.84 months [95%-CI: 4.67–9.70], HR: 2.77 [95%-CI: 1.40–5.47], Cox regression *p* = 0.003, Fig. 4C) and OS (4.78 months [95%-CI: 0.72–8.84] vs. 14.10 months [95%-CI:12.06–19.86], HR: 3.18 [95%-CI: 1.60–6.30], Cox regression *p* = 0.001, Fig. 4D) was also found for EM AML patients with mutated *IKZF1* compared to wildtype EM AML patients. Again, multivariable analysis adjusting for age and ELN2017 risk groups revealed mutated *IKZF1* as an independent marker of reduced EFS (HR: 2.05 [95%-CI: 1.02–4.13], Cox regression *p* = 0.044, Additional file 1: Table S6) and OS (HR: 2.63 [95%-CI: 1.29–5.39], Cox regression *p* = 0.008, Additional file 1: table S7). No significant differences in survival times of EM AML patients were found for mutations of *NPM1*, *FLT3-ITD*, *PTPN11*, *ASXL1*, *RUNX1*, or *CEBPA*.

In our cohort, 573 (36.20%) of patients received an allogeneic hematopoietic cell transplantation, of whom 66 (11.52%) had EM AML. In a post-transplant setting, relapse rates did not differ between patients with and without EM AML (OR: 1.00 [95%-CI: 0.60–1.68], *p* = 0.992]. In contrast to the overall patient cohort, survival rates of patients harboring EM AML compared to those who did not present with EM did not differ (Table 4).

**Table 4:** Survival times of patients with or without extramedullary manifestations who received allogeneic hematopoietic stem cell transplantation

Survival times of patients who received HCT	EM AML	Non-EM AML	Hazard ratio	Cox regression *p*-value
Event-free survival	9.8 [6.7–14.8]	10.7 [9.1–13.2]	0.98 [0.73–1.33]	0.910
Relapse-free survival	13.0 [8.2–37.5]	16.3 [12.7–20.0]	0.99 [0.72–1.38]	0.975
Overall survival	30.8 [18.2–54.2]	72.1 [48.7–103.7]	1.29 [0.93–1.80]	0.130

Survival times for AML patients with and without extramedullary manifestations (EM) that received allogeneic hematopoietic cell transplantation (HCT, *n* = 573, 36.20%) are shown. Survival times in months. Cox-proportional hazard models were used to obtain univariable hazard ratios. Brackets show 95%-confidence intervals

Further, we analyzed patients harboring EM by ELN2017 subgroup with regard to the impact of HCT. For EM AML patients in the ELN2017 favorable risk group, we found no significant difference between patients who received or who did not receive HCT with respect to EFS, RFS, and OS. For EM AML patients in the ELN2017 intermediate risk group, RFS did not differ between patients who received or did not receive HCT. However, EFS (median 9.1 vs. 3.6, HR: 0.55 [95%-CI: 0.32–0.98], Cox regression *p* = 0.043) and OS (median 44.1 vs. 7.5 months, HR: 0.40 [95%-CI: 0.21–0.77], Cox regression *p* = 0.006) were significantly prolonged for ELN intermediate EM AML patients who received HCT. Finally for EM AML patients in the ELN2017 adverse risk group, again both EFS (median 7.4 vs. 1.2 months, HR: 0.48 [95%-CI: 0.27–0.85], Cox regression *p* = 0.013) and OS (median 21.0 vs. 7.1 months, HR: 0.31 [95%-CI: 0.17–0.58], Cox regression *p* < 0.001) were significantly longer for patients who received HCT compared to patients who did not receive HCT while RFS did not differ. Table 5 summarizes the impact of HCT on survival times for EM AML patients according to ELN2017 risk groups.

**Table 5:** Impact of hematopoietic stem cell transplantation in for AML patients with extramedullary manifestations in ELN2017 groups

Survival times of EM AML patients according to ELN2017 groups	HCT	no HCT	Hazard ratio	Cox regression *p*-value
ELN2017 favorable	*n* = 20	*n* = 61		
Event-free survival	13.5 [4.8-n.r.]	12.6 [7.8–27.5]	0.86 [0.45–1.65]	0.659
Relapse-free survival	29.0 [7.5-n.r.]	43.6 [12.6-n.r.]	1.35 [0.66–2.78]	0.410
Overall survival	54.0 [11.0-n.r.]	27.2 [13.6-n.r.]	0.81 [0.41–1.59]	0.537
ELN2017 intermediate	*n* = 22	*n* = 55		
Event-free survival	9.1 [3.7–44.1]	3.6 [1.8–7.5]	0.55 [0.32–0.98]	**0.043**
Relapse-free survival	10.8 [4.6-n.r.]	8.4 [2.1–14.4]	0.69 [0.35–1.35]	0.277
Overall survival	44.1 [9.1-n.r.]	7.5 [3.6–13.1]	0.40 [0.21–0.77]	**0.006**
ELN2017 adverse	*n* = 23	*n* = 33		
Event-free survival	7.4 [1.1–13.6]	1.2 [0.3–3.0]	0.48 [0.27–0.85]	**0.013**
Relapse-free survival	11.6 [4.2–44.0]	6.0 [2.3–9.8]	0.51 [0.24–1.08]	0.077
Overall survival	21.0 [13.6–48.4]	7.1 [3.1–9.2]	0.31 [0.17–0.58]	** < 0.001**

Survival times for AML patients harboring extramedullary manifestations (EM) are compared for patients receiving or not receiving hematopoietic stem cell transplantation (HCT). Survival times in months. Cox-proportional hazard models were used to obtain univariable hazard ratios. Brackets show 95%-confidence intervals. n.r. = not reached (due to small sample sizes and thus limited numbers of events within the ELN subgroups of EM AML patients who underwent HCT not all upper confidence interval limits can be estimated)

## Discussion

We analyzed a large cohort of newly diagnosed AML patients, 14% of whom harbored EM based on clinical and/or histological diagnostics. Regarding clinical presentation, AML with EM was associated with significantly higher WBC, PBB, BMB and LDH at diagnosis. With the exception of two patients, all EM AML patients had concurrent bone marrow involvement. An increase in bone marrow infiltration as well as an increase in peripheral blood involvement increased the odds of EM. However, it has to be noted that our analysis likely underestimates the incidence of myeloid sarcoma, i. e. EM without infiltration of the bone marrow, as these cases were not eligible to be included in the four previous clinical trials pooled for this analysis and the cases stem from the SAL bioregistry. Previous reports have suggested a higher incidence of EM AML in FAB-M2, -M4, and -M5 [22]. In our sample, EM were significantly more frequent in patients with FAB-M5a and FAB-M5b while FAB-M2, -M6, and -4 rarely presented with EM.

For cytogenetics, previous reports in smaller cohorts and case series have linked the occurrence of EM AML with cytogenetic aberrations like t(8;21) [23, 24], trisomy 8 [25, 26] and inv [16, 27]. In our analysis, we did not find a statistically significant association between EM AML and either t(8;21) or inv [16] and for trisomy 8 there was even a trend for a lower prevalence of EM AML. Regarding molecular genetics, mutations of *NPM1* and *FLT3*-ITD have been associated with EM AML. For *NPM1*, Ovcharenko et al. [28] report 13 out of 15 EM AML patients to harbor mutated *NPM1* in a cohort of 89 patients with AML, Falini et al. [29] reported mutated *NPM1* in 14% of 181 EM AML samples, and Döhner et al. [30] reported a correlation of mutated *NPM1* and gingival hyperplasia. Ansari-Lari et al. [31] also identified *FLT3*-ITD in 15% of EM AML samples. Further, recent studies employing next-generation sequencing described the presence of mutations of *KIT, WT1, TET1, ASXL1, SF3B1* and *EZH2* [32] as well as *NPM1, NRAS*, and *DNMT3A* [33] in EM AML. In line with these previous findings, we found significantly increased odds for the presence of EM AML for mutations of *NPM1* and *FLT3*-ITD while higher *FLT3*-ITD ratio was associated with higher odds of EM AML. Additionally, we found mutations of *PTPN11* to be significantly associated with the presence of EM AML while the odds for EM AML were significantly decreased in *IDH2*- or *CEBPA*-mutated AML. *PTPN11* has recently been described as an independent marker of poor outcome in AML [34, 35] and has been associated with EM [36]. *PTPN11* encodes for the Src homology region 2 domain-containing phosphatase-2 (SHP-2) which functions as a signal enhancer for cell growth and differentiation downstream of numerous intracellular pathways including RAS/ERK/MAPK, JAK/STAT as well as PI3K/AKT and FLT3 signaling therefore playing a critical role in leukemogenesis [37–41]. However, its role in the formation of EM is insufficiently defined and further investigations are warranted to shed light on the mechanisms of how disrupted SHP2 signaling drives EM formation. While mutations of *CEBPA* were associated with significantly decreased odds of EM AML in our cohort, this effect was not seen individually in biallelic mutations of *CEBPA*, *CEBPA-TAD* or *CEBPA-bZIP*, rendering the mechanism unclear and thereby calling for more detailed investigations as subtypes of *CEBPA* have been reported to show differences in clinical outcomes [18].

With respect to clinical outcome, the impact of EM in AML is still controversial. In a retrospective analysis of 3240 AML patients, Ganzel et al. [42] found 23.7% to bear EM, however EM status was not associated with differences in survival between EM and non-EM AML. Fernandez et al. [43] found no significant associations between CR rate and disease-free survival for EM status. Accordingly, Agis et al. [44] reported no significant associations between leukemia cutis and survival in 381 AML patients and Ganzel et al. [45] did not find a difference in outcome for AML patients with CNS involvement. In contrast, in our analysis we found EM to be an independent marker of poor outcome. EM were associated with significantly decreased odds of achieving CR with standard intensive therapy while the odds of ED_30_ were significantly increased. Although EFS and RFS did not differ between patients with EM AML and non-EM AML when all patients including clinical and histological diagnosis of EM where investigated (Fig. 2A–C), we found OS to be significantly decreased. This finding was confirmed in multivariable analysis including age and ELN2017 risk categories. Interestingly, when we excluded patients for whom EM has been only diagnosed clinically and only included patients for whom EM has been confirmed by histology, not only was EFS also affected, but the effect on decreased OS became even more pronounced (Fig. 2D–F). For these 38 patients compared to non-EM patients (and excluding patients for whom only clinical diagnosis of EM was available), we found significantly decreased CR rates as well as significantly decreased EFS and OS both in uni- and multivariable analysis. Strikingly, by excluding patients with only clinical diagnosis of EM AML, effect sizes for decreased CR, EFS and OS became notably larger compared to our previous analysis when clinical and histological EM AML were combined suggesting that patients in the clinical EM AML group may have been false positive (possibly patients with gingival hyperplasia). Another possibility could be reduced salvageability for patients bearing EM resulting in comparable EFS but worse OS, however, this is speculative as data on response to salvage treatments except for HCT are not available. For the majority of our cohort, EM status was determined clinically at the discretion of the treating physician (which includes cases of gingival hyperplasia) and only in 38 patients biomaterial from the affected EM sites was available for histopathologic assessment. It is therefore conceivable that there were also patients with EM that have not been detected by clinical judgement alone. This discrepancy between histologically confirmed and clinically diagnosed EM could be an explanation as to why several previous reports showed discrepant rates of EM in AML and did not identify a difference in outcome for EM AML [30, 42, 43]. Therefore, the dilemma of detecting EM in AML is twofold with a considerable margin of error on both sides of the spectrum: On the one hand, in both clinical trials (including ours) the frequency of EM is likely underestimated due to insufficient screening via imaging such as PET and insufficient histological confirmation of potential EM sites which likely contributes to a substantial false negative rate. On the other hand, clinical diagnosis of EM is subjective and often performed (as in our study) at the discretion of the treating physician. This is why clinical symptoms can be misinterpreted as EM if no histological confirmation is obtained. This can potentially lead to false positives. In our analysis, clinical diagnosis of EM was followed by assessment of regression of these lesions after induction therapy according to ELN2017 recommendations for CR assessment and the label ‘CR achieved’ was only given for patients with complete regression of any clinically suspected EM lesions. However, this does not fully exclude potential false positives as misinterpreted lesions may have regressed due to other causes. Hence, it seems reasonable for future studies to focus on histologically confirmed cases of EM AML. However, due to potential clinical complications involved in taking a biopsy in AML patients with increased risk of bleeding acquiring tissue for histopathological confirmation of EM will likely not be possible in routine practice and even in the context of clinical trials it has to be evaluated on a case-by-case basis as patient safety outweighs potential knowledge gains. Given current limitations in detection mechanisms of EM via imaging or biopsy in addition to medical and ethical restrictions regarding a cost-to-benefit wager, it is questionable whether a fully representative study of the distribution of EM between sites with histological confirmation is actually obtainable given that not all suspected sites in all patients can be sampled and examined histologically due to safety reasons. The difference in outcomes could stem from the discrepancy between CR in the bone marrow while extramedullary sites may persist as can be shown by ^18^FDG-PET [9] and thus drive relapse. Hence, these patients constitute a special group at risk of undertreatment as they are falsely considered to having achieved CR while they actually could benefit from therapy intensification due to refractory EM that could only be detected by including more rigorous screening via imaging upon treatment response assessment. While for our cohort no data for measurable residual disease (MRD) was available, a comparative investigation of peripheral blood MRD levels for patients with or without EM in AML could provide additional insights for relapse monitoring in cases with bone marrow CR. Due to the rarity of EM AML, studies on risk factors for patients with EM are scarce. Regarding risk stratification for EM AML, Ullmann et al. reported OS to be associated with certain cytogenetic aberrations [10], while no such associations were found in our sample. Nevertheless, in univariable and multivariable analysis adjusting for age and ELN2017 risk category, we found both *TP53* and *IKZF1* mutations to be independently associated with reduced EFS and OS. While mutated *TP53* is an established marker of adverse risk in AML in general [46], the role of *IKZF1* alterations is far less well understood in AML than it is in acute lymphoblastic leukemia [47, 48]. Interestingly, *IKZF1*-alterations have been recently identified in patients with blastic plasmocytoid dendritic cell neoplasms, a rare hematological disease associated with cutaneous and solid organ infiltration and poor prognosis [49]. Further investigation is warranted to illuminate its role in both medullary and extramedullary disease. Consensually, patients with EM AML and high-risk cytogenetics are candidates for allogeneic HCT [50] and relapse rates as well as post-transplant survival have been reported not to differ [5, 51–53]. In line with these findings, in our analysis relapse rates as well as median EFS, RFS and OS did not differ between patients with or without EM who received allogeneic HCT. This is of special interest, as the existence of sanctuary sites and the potential risk of immune escape and EM has been described in patients with AML relapsing after allogeneic HCT [54]. Possibly, the choice of radiotherapy as conditioning therapy may be indicated in patients with a history of EM [4]. Hence, allogeneic HCT may be considered in patients with EM AML in the absence of other risk-defining cytogenetic or molecular markers.

## Conclusion

We analyzed a large cohort of AML patients according to the molecular and cytogenetic profiles of EM AML and its impact on survival. We found EM AML to be significantly associated with AML-M5 as well as mutations of *NPM1*, *PTPN11* and *FLT3*-ITD while it was less frequent in AML with mutated *IDH2* or *CEBPA*. For patients harboring EM AML, mutations in *TP53* and *IKZF1* were found to be independently associated with poor outcome. In multivariable analysis, EM represented an independent marker of reduced OS. However, survival did not differ between patients with or without EM who received HCT suggesting an important role of allogeneic transplantation in the management of EM AML.

## Supplementary Information

**Additional file 1:** Impact of continuous variables on odds of EM manifestation and  multivariable analysis for outcome.

## Data Availability

The datasets analyzed during the current study are available from the corresponding author on reasonable request.

## Abbreviations

AML: Acute myeloid leukemia
sAML: Secondary acute myeloid leukemia
tAML: Therapy-associated acute myeloid leukemia
BMB: Bone marrow blast count
EFS: Event-free survival
EM: Extramedullary manifestations
FISH: Fluorescence in situ hybridization
HCT: Hematopoietic cell transplantation
MRD: Measurable residual disease
PBB: Peripheral blood blast count
RFS: Relapse-free survival
OS: Overall survival
WBC: White blood cell count
