# Supplementary material for: Molecular profiling and clinical implications of patients with acute myeloid leukemia and extramedullary manifestations
Source: J Hematol Oncol. 2022 May 13;15:60. doi: 10.1186/s13045-022-01267-7 (PMC9107142; doi:10.1186/s13045-022-01267-7)
Supplement: Supplementary file 1 — Additional file 1: Impact of continuous variables on odds of EM manifestation and multivariable analysis for outcome. [file 13045_2022_1267_MOESM1_ESM.docx]

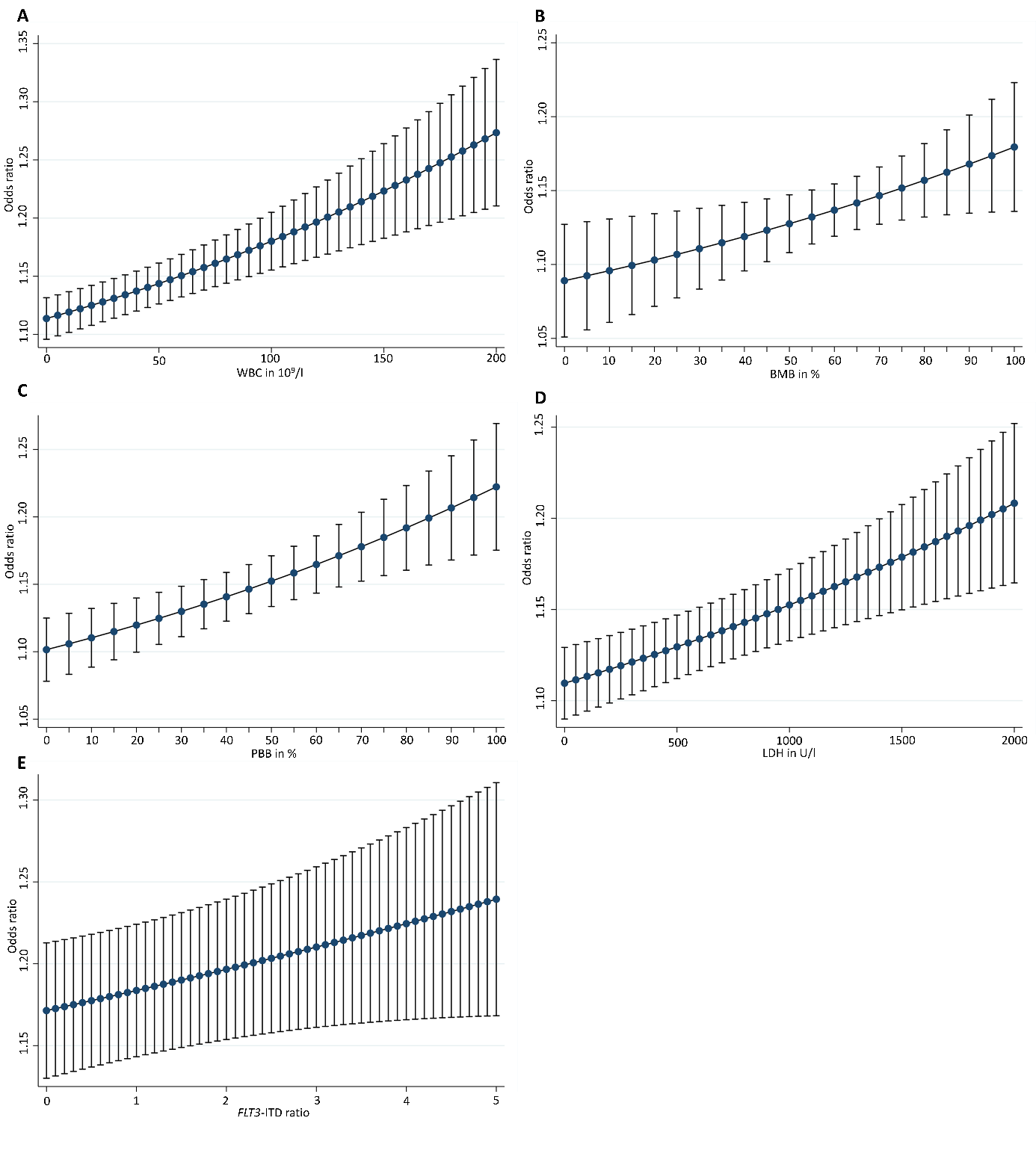


**Figure S1. Impact of continuous parameters on the presence of extramedullary manifestations in AML**

An increase in white blood cell count (WBC, **A**), bone marrow blast count (BMB, **B**), peripheral blood blast count (PBB, **C**) as well as LDH (**D**) and *FLT3*-ITD ratio (**E**) corresponded to an increase in the odds of the presence of extramedullary manifestations in AML.

| **Variable name** | **Hazard ratio [95%-CI]** | ***p*-value** |
| --- | --- | --- |
| extramedullary manifestations | 1.43 [1.21-1.70] | **<0.001** |
| age | 1.04 [1.03-1.04] | **<0.001** |
| ELN2017 favorable risk | 0.63 [0.48-0.82] | **0.001** |
| ELN2017 intermediate risk | 1.04 [0.81-1.35] | 0.743 |
| ELN2017 adverse risk | 1.41 [1.10-1.83] | **0.008** |

**Supplementary Table 1.** Multivariable analysis for overall survival for the entire cohort

| **Variable name** | **Hazard ratio [95%-CI]** | ***p*-value** |
| --- | --- | --- |
| extramedullary manifestations | 1.44 [1.01-2.05] | **0.042** |
| age | 1.03 [1.02-1.03] | **<0.001** |
| ELN2017 favorable risk | 0.56 [0.43-0.72] | **<0.001** |
| ELN2017 intermediate risk | 1.00 [0.79-1.29] | 0.959 |
| ELN2017 adverse risk | 1.44 [1.13-1.83] | **0.003** |

**Supplementary Table 2.** Multivariable analysis for event-free survival for the comparison of patients with histologically confirmed extramedullary manifestations (EM) and patients without EM (excluding patients with only clinical diagnosis of EM)

| **Variable name** | **Hazard ratio [95%-CI]** | ***p*-value** |
| --- | --- | --- |
| extramedullary manifestations | 1.67 [1.67-2.40] | **0.005** |
| age | 1.04 [1.03-1.04] | **<0.001** |
| ELN2017 favorable risk | 0.62 [0.46-0.82] | **0.001** |
| ELN2017 intermediate risk | 1.02 [0.78-1.35] | 0.865 |
| ELN2017 adverse risk | 1.38 [1.06-1.81] | **0.019** |

**Supplementary Table 3.** Multivariable analysis for overall survival for the comparison of patients with histologically confirmed extramedullary manifestations (EM) and patients without EM (excluding patients with only clinical diagnosis of EM)

| **Variable name** | **Hazard ratio [95%-CI]** | ***p*-value** |
| --- | --- | --- |
| mutated *TP53* | 4.45 [1.94-10.20] | **<0.001** |
| age | 1.03 [1.02-1.05] | **<0.001** |
| ELN2017 favorable risk | 0.57 [0.29-1.14] | 0.115 |
| ELN2017 intermediate risk | 1.09 [0.55-2.15] | 0.803 |
| ELN2017 adverse risk | 1.53 [0.76-3.11] | 0.234 |

**Supplementary Table 4.** Multivariable analysis for event-free survival for patients with extramedullary manifestations with regard to the impact of mutated *TP53*

| **Variable name** | **Hazard ratio [95%-CI]** | ***p*-value** |
| --- | --- | --- |
| mutated *TP53* | 2.48 [1.11-5.52] | **0.026** |
| age | 1.03 [1.01-1.04] | **<0.001** |
| ELN2017 favorable risk | 0.60 [0.30-1.21] | 0.152 |
| ELN2017 intermediate risk | 1.04 [0.52-2.07] | 0.916 |
| ELN2017 adverse risk | 1.22 [0.60-2.49] | 0.575 |

**Supplementary Table 5.** Multivariable analysis for overall survival for patients with extramedullary manifestations with regard to the impact of mutated *TP53*

| **Variable name** | **Hazard ratio [95%-CI]** | ***p*-value** |
| --- | --- | --- |
| mutated *IKFZ1* | 2.05 [1.02-4.13] | **0.044** |
| age | 1.03 [1.02-1.05] | **<0.001** |
| ELN2017 favorable risk | 0.60 [0.30-1.20] | 0.147 |
| ELN2017 intermediate risk | 1.23 [0.62-2.44] | 0.560 |
| ELN2017 adverse risk | 1.62 [0.80-3.29] | 0.179 |

**Supplementary Table 6.** Multivariable analysis for event-free survival for patients with extramedullary manifestations with regard to the impact of mutated *IKZF1*

| **Variable name** | **Hazard ratio [95%-CI]** | ***p*-value** |
| --- | --- | --- |
| mutated *IKFZ1* | 2.63 [1.29-5.39] | **0.008** |
| age | 1.03 [1.01-1.04] | **<0.001** |
| ELN2017 favorable risk | 0.62 [0.30-1.24] | 0.176 |
| ELN2017 intermediate risk | 1.17 [0.58-2.35] | 0.660 |
| ELN2017 adverse risk | 1.19 [0.59-2.44] | 0.625 |

**Supplementary Table 7.** Multivariable analysis for overall survival for patients with extramedullary manifestations with regard to the impact of mutated *IKZF1*
